# Virus-Induced Epilepsy vs. Epilepsy Patients Acquiring Viral Infection: Unravelling the Complex Relationship for Precision Treatment

**DOI:** 10.3390/ijms25073730

**Published:** 2024-03-27

**Authors:** Bárbara Costa, Nuno Vale

**Affiliations:** 1 PerMed Research Group, Center for Health Technology and Services Research (CINTESIS), Rua Doutor Plácido da Costa, s/n, 4200-450 Porto, Portugal; b.c.211297@gmail.com; 2 CINTESIS@RISE, Faculty of Medicine, University of Porto, Alameda Professor Hernâni Monteiro, 4200-319 Porto, Portugal; 3 Department of Community Medicine, Information and Health Decision Sciences (MEDCIDS), Faculty of Medicine, University of Porto, Rua Doutor Plácido da Costa, s/n, 4200-450 Porto, Portugal

**Keywords:** virus-induced epilepsy, epilepsy patients, neuroinflammation, anticonvulsant drugs, seizure susceptibility, anticonvulsant drug pharmacokinetics, clinical management

## Abstract

The intricate relationship between viruses and epilepsy involves a bidirectional interaction. Certain viruses can induce epilepsy by infecting the brain, leading to inflammation, damage, or abnormal electrical activity. Conversely, epilepsy patients may be more susceptible to viral infections due to factors, such as compromised immune systems, anticonvulsant drugs, or surgical interventions. Neuroinflammation, a common factor in both scenarios, exhibits onset, duration, intensity, and consequence variations. It can modulate epileptogenesis, increase seizure susceptibility, and impact anticonvulsant drug pharmacokinetics, immune system function, and brain physiology. Viral infections significantly impact the clinical management of epilepsy patients, necessitating a multidisciplinary approach encompassing diagnosis, prevention, and treatment of both conditions. We delved into the dual dynamics of viruses inducing epilepsy and epilepsy patients acquiring viruses, examining the unique features of each case. For virus-induced epilepsy, we specify virus types, elucidate mechanisms of epilepsy induction, emphasize neuroinflammation’s impact, and analyze its effects on anticonvulsant drug pharmacokinetics. Conversely, in epilepsy patients acquiring viruses, we detail the acquired virus, its interaction with existing epilepsy, neuroinflammation effects, and changes in anticonvulsant drug pharmacokinetics. Understanding this interplay advances precision therapies for epilepsy during viral infections, providing mechanistic insights, identifying biomarkers and therapeutic targets, and supporting optimized dosing regimens. However, further studies are crucial to validate tools, discover new biomarkers and therapeutic targets, and evaluate targeted therapy safety and efficacy in diverse epilepsy and viral infection scenarios.

## 1. Introduction

Viruses and epilepsy have a complex and bidirectional relationship. On one hand, some viruses can cause epilepsy by infecting the brain and triggering inflammation, damage, or abnormal electrical activity. On the other hand, some epilepsy patients are more prone to acquiring viral infections due to their impaired immune system, anticonvulsant drugs, or surgical interventions [1]. Understanding the distinctions between these two perspectives is crucial for developing tailored therapeutic approaches that can prevent or treat viral-induced epilepsy and protect epilepsy patients from viral complications.

**Figure 1:**
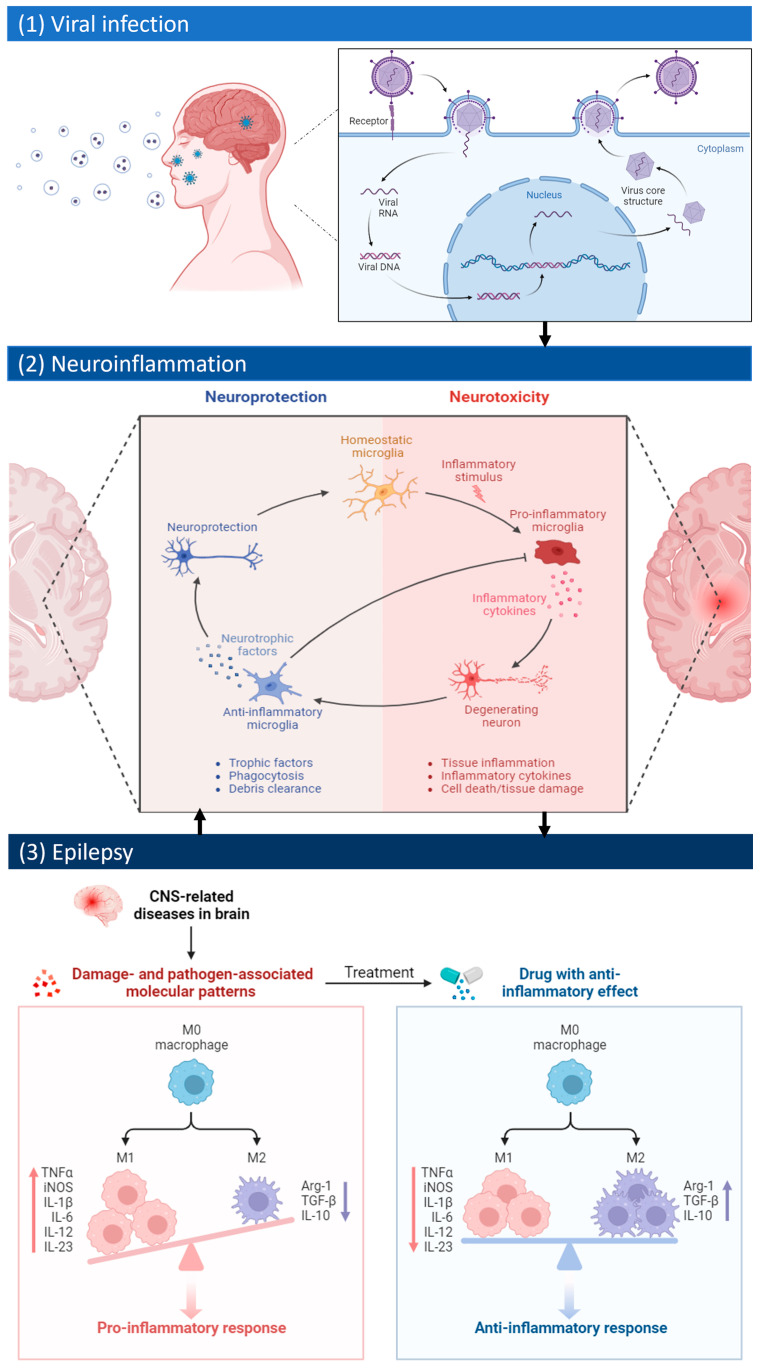
Illustrative image of the interplay of viral infection, epilepsy, neuroinflammation and treatment management of disease. Neuroinflammation can be induced under several conditions, including viral infections. The main immune cells in the brain, microglia, play a pivotal role in neuroinflammation by responding to invading pathogens (viral DNA/RNA) through toll-like receptors (TLRs). Chronic activation of microglia caused by sustained viral infection leads to the persistent release of pro-inflammatory molecules. This is different from their beneficial functions under physiological conditions. The release of these pro-inflammatory factors, including tumor necrosis factor-α (TNFα) and interleukin-1β (IL-1β), free radicals such as nitric oxide (NO) and superoxide, is initially a defensive strategy of the immune system. However, sustained exposure of neurons to these inflammatory factors can result in neuronal dysfunction and cell degeneration, contributing to the pathogenesis of aging-related neurodegenerative disease. Viral infection in the CNS is a common cause of seizures and epilepsy. Sustained exposure of neurons to these inflammatory conditions can result in neuronal dysfunction and cell degeneration, contributing to the pathogenesis of several neurological disorders. Neuroprotection is a therapeutic strategy aimed at preventing or reducing damage to the nervous system caused by various pathological conditions, including neuroinflammation. Anti-inflammatory drugs can be used to treat CNS-related diseases by reducing the release of pro-inflammatory molecules, including TNFα and IL-1β, and free radicals such as NO and superoxide, which can contribute to neuronal dysfunction and cell degeneration. Adapted from BioRender.com, accessed on 7 December 2023.

In the context of viruses inducing epilepsy, certain pathogens possess the ability to directly infiltrate the central nervous system, triggering a cascade of events that can lead to seizures (Figure 1) [2,3]. Understanding the mechanisms by which these viruses navigate the neural environment, incite neuroinflammation, and contribute to the epileptogenic process is fundamental [4]. This perspective demands a focus on specific viral characteristics, routes of neuroinvasion, and the ensuing inflammatory responses that shape the manifestation of epilepsy [3]. On the other hand, individuals with pre-existing epilepsy face a distinct set of challenges when encountering viral infections. Factors, such as altered immune responses, the influence of anticonvulsant drugs on the immune system, or the impact of seizures on overall health, contribute to increased susceptibility to viral pathogens [5,6]. Acute symptomatic seizures can occur in the context of almost all types of acute central nervous system (CNS) viral infection, and patients with pre-existing epilepsy may experience more frequent seizures during viral infection [7,8]. Additionally, viral infections can trigger status epilepticus, which is a medical emergency that requires prompt treatment [9]. Late unprovoked seizures and epilepsy may not be frequent after viral infection of the CNS [3].

The importance of distinguishing between these perspectives lies in the potential for tailored therapeutic interventions. Precision medicine approaches can only be successful when grounded in a comprehensive understanding of the specific dynamics at play. Differentiating between cases where viruses function as primary instigators of epilepsy and instances where individuals with epilepsy become vulnerable to viral infections informs targeted treatment strategies, optimizing outcomes and minimizing potential risks associated with therapeutic interventions (Table 1).

Epilepsy is a chronic neurological disorder that affects about 50 million people worldwide, making it one of the most common neurological diseases globally [10]. It is characterized by recurrent and unpredictable seizures and sudden bursts of electrical activity in the brain. These seizures can manifest in various forms, from momentary lapses in awareness to intense convulsions [11]. The underlying causes of epilepsy are diverse, ranging from genetic factors and brain injuries to infections and structural abnormalities [12]. Viral infections are one of the potential causes of seizures and epilepsy [13,14]. Viruses can intricately navigate various pathways to breach the protective barriers guarding the brain [15]. Viruses can enter the brain through different routes, such as the bloodstream, the olfactory nerve, or the peripheral nerves, and infect and damage neurons, glial cells, or endothelial cells. Neurotropic viruses can travel from the point of entry in the body to the CNS, where they can potentially infect local cells and induce neurological disorders. These pathways may be linked together, with one perhaps initiating others. Whether they can coexist depends on which pathway is more important and contributes more to neuroinvasion [15].

Viral infections can induce neuroinflammation, which is a process that involves the activation of immune cells and the release of inflammatory mediators in the brain [16,17]. Neuroinflammation can have beneficial and detrimental effects on the brain, depending on the type, duration, and intensity of the inflammatory response [18]. Consequently, viral infections are among the most prevalent causes of epilepsy, particularly when they impact the CNS, as they can induce tissue inflammation, damage, and scarring in brain tissue, which can change the electrical activity of neurons and produce seizures [19]. Some of the common CNS viral infections that can cause epilepsy are herpes simplex virus (HSV), HIV, rabies virus, West Nile virus, and Japanese encephalitis virus [20]. Notably, amid the COVID-19 pandemic, the coronavirus was associated with severe outcomes, including rare instances of epilepsy and stroke. However, it is essential to recognize that more common neurological manifestations of COVID-19 infection encompass symptoms, such as delirium, confusion, headache, and the loss of sense of smell and taste [21,22]. In any case, children seem to be particularly susceptible to experiencing seizures and developing epilepsy following a COVID-19 infection, serving as an additional incentive to avoid COVID-19 transmission among pediatric populations [22,23]. Neuroinflammation can contribute to the development and progression of epilepsy by altering the balance between excitatory and inhibitory neurotransmission, increasing neuronal vulnerability, and facilitating the formation of epileptic foci [24]. Neuroinflammation is also a consequence of epileptic seizures (Figure 1), as seizures can activate glial cells, release pro-inflammatory cytokines, and disrupt the blood–brain barrier [25,26,27].

**Figure 2:**
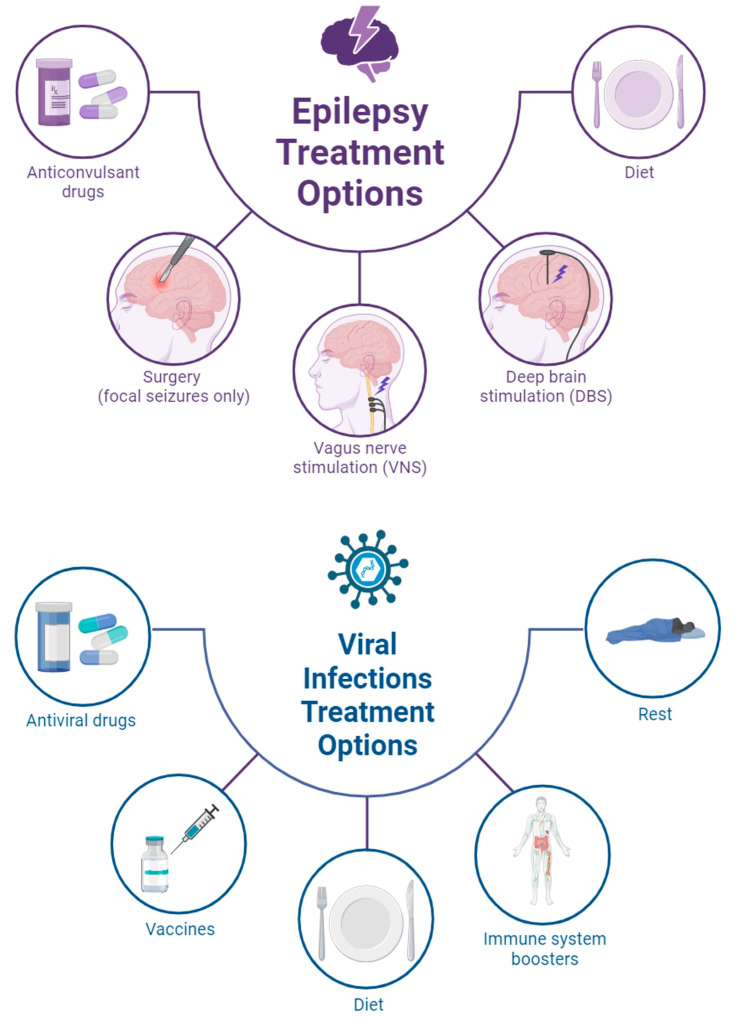
Schematic representation of the treatment options for viral infection and epilepsy. Anticonvulsant drugs are the most common treatment for epilepsy. Some patients may require surgery to remove a small part of the brain that is causing the seizures, or a small electrical device may be used inside the body to help control seizures. A special diet (ketogenic diet) may help control seizures. Antiviral drugs are available to treat some viral infections. Some medications, such as interferons and cytokines, even mimic the body’s natural immune system stimulation signals. Vaccines are usually given to prevent infection, but some can also be used to treat people who have recently been infected. Both types of conditions often require a long-term treatment approach and may require adjustments in treatment over time. Treatments for both conditions often focus on managing symptoms and improving the patient’s quality of life. Adapted from BioRender.com, accessed on 7 December 2023.

The interplay between viral infections and neuroinflammation is a complex and dynamic phenomenon that may influence the diagnosis, treatment, and prognosis of epilepsy. In this intricate landscape, the use of anticonvulsant and antiviral therapies becomes pivotal in managing the dual challenges posed by both viral infections and epilepsy. While anticonvulsants play a central role in controlling seizures and improving the quality of life for individuals with epilepsy, integrating antiviral therapies becomes equally essential when addressing cases where viral infections contribute to or trigger epileptic episodes (Figure 2). The synergy between these therapeutic approaches is crucial for developing comprehensive and tailored strategies that not only target the symptoms of epilepsy but also address the underlying viral causes, thereby optimizing patient outcomes [28,29].

Moreover, viral infections and neuroinflammation can profoundly influence the pharmacokinetics of anticonvulsants by modulating the expression and activity of enzymes and transporters crucial for their metabolism and transport [30]. The cytochrome P450 (CYP) system, a key player in the metabolism of several anticonvulsants, such as valproic acid, phenytoin, carbamazepine, and phenobarbital, is notably impacted during viral infections, potentially leading to increased metabolism and reduced plasma levels of these medications [3]. This, in turn, may compromise seizure control and elevate the risk of breakthrough seizures [31,32,33]. Conversely, anticonvulsants like lamotrigine and levetiracetam, which are not extensively metabolized by CYP450 enzymes [34], might exhibit less susceptibility to alterations in plasma levels during viral infections. Additionally, the potential interaction of anticonvulsants with medications employed for treating viral infections, such as antivirals, antibiotics, or anti-inflammatory drugs, underscores the need to monitor anticonvulsant plasma levels and adjust doses accordingly [3]. Since viral infections can induce seizures and epilepsy through various mechanisms [32], these mechanisms may impact the efficacy and safety of anticonvulsants, necessitating tailored therapeutic strategies. Some anticonvulsants may exhibit anti-inflammatory or neuroprotective effects, while others may exacerbate inflammation or neuronal damage. Hence, selecting an appropriate anticonvulsant should be guided by the specific viral infection type, seizure characteristics, and the patient’s overall condition [32]. The interaction between anticonvulsants and antivirals carries significant clinical implications, encompassing potential toxicity, compromised seizure control, and incomplete viral suppression [33,34]. Noteworthy examples include the potential for carbamazepine serum concentrations to rise with concurrent ritonavir, leading to toxicity, and the likelihood of phenytoin serum concentrations decreasing with nelfinavir, resulting in recurrent seizures [33]. Vigilant monitoring of these interactions is crucial, considering newer anticonvulsant agents as potential alternatives. Moreover, the use of antiviral drugs, particularly nucleoside analogs, introduces additional complexities with their adverse effects and potential drug interactions, necessitating careful evaluation when combined with anticonvulsants [35,36].

The insights gained from understanding these intricate interactions are not only pertinent to epileptic patients but also extend to individuals experiencing viral infections that may potentially lead to epilepsy. Furthermore, this understanding opens up avenues for exploring innovative approaches to developing new anticonvulsants or adjunct therapies specifically designed to target inflammatory processes, thereby offering potential improvements in epilepsy outcomes. By navigating the complexities of viral infections and epilepsy with this dual perspective, we not only enhance our comprehension of these conditions but also pave the way for the development of precision therapeutic frameworks tailored to the unique needs of individuals.

## 2. Virus-Inducing Epilepsy

Virus-induced epilepsy is when viral infection in the CNS causes seizures and epilepsy, either during the acute phase of infection or later as a consequence of brain damage or inflammation [37]. The prevalence of epilepsy in individuals with viral infections varies depending on the type of virus, the severity of the infection, the location of the brain lesion, and other risk factors [3,38]. Several viruses can infect the brain and cause inflammation, damage, or abnormal electrical activity, leading to epilepsy. For instance, the prevalence of seizures in patients with HIV is substantially higher than in the general population, with advanced HIV and opportunistic infections being major risk factors [39]. Similarly, viral encephalitis, particularly herpes-associated encephalitis, can significantly increase the risk of epilepsy [40]. In children with viral encephalitis, the risk factors for post-viral encephalitic epilepsy include repetitive seizures, clinical seizures detected in EEG, and status epilepticus [41]. However, the prevalence and incidence of epilepsy do not differ by age group, sex, or study quality [42]. The prevalence of epilepsy after viral infection can change over time, with some individuals experiencing late-onset epilepsy while others may achieve seizure remission. Fujita et al. reported cases of intractable childhood epilepsy improving following acute viral infection, with some patients becoming seizure-free for several years [43]. However, there is an increased risk of developing epilepsy, particularly after viral encephalitis, which can lead to severe neurological sequelae [44].

Unfortunately, the majority of the disease burden is borne by the developing world [45], which results in limited resources being allocated to deciphering how cestode brain infection ultimately triggers epilepsy as asserted by Steyn [46]. This missed opportunity not only hinders the development of much-needed therapeutics for epilepsy in this specific context but also impedes the discovery of innovative anticonvulsant strategies with broader applicability in the treatment of epilepsy on a global scale [46,47]. Other examples include cerebral malaria, caused by Plasmodium parasites, leading to vessel blockage in the brain, commonly affecting children and pregnant women and resulting in seizures and death [48,49]. TORCH infections transmitted during pregnancy include toxoplasmosis, syphilis, and herpes simplex, leading to congenital abnormalities and epilepsy [50,51] Bacterial meningitis, caused by various bacteria, can induce seizures and epilepsy if untreated [52,53]. According to the available evidence, COVID-19 infection does not directly induce epilepsy in most cases. However, a study published by Taquet et al. found that there is a low absolute risk of epilepsy and seizures after COVID-19 infection. The study showed that people who had COVID-19 were not at higher risk of developing epilepsy or seizures than those who had not had COVID-19 [54]. However, the study did not rule out the possibility that COVID-19 could contribute to seizures and epileptogenesis in some cases. The study also suggested that stroke, which can be a potential consequence of COVID-19, may be the main cause of COVID-19-related seizures or epilepsy. However, there is an increased risk of developing seizures in the six months following an infection of COVID-19. The possible mechanisms of epilepsy induced by COVID-19 include cytokine storm and secondary seizures. Secondary seizures may be initiated after strokes, electrolyte imbalance, increased oxidative stress, and mitochondrial dysfunction in COVID-19 patients. People with epilepsy may also experience more seizures during the pandemic due to stress and difficulty accessing healthcare [55].

The COVID-19 pandemic presented unique challenges in the field of epilepsy and has led researchers to explore the relationship between viruses and epilepsy. Current findings provide a basis for understanding the mechanisms of how viruses may affect epilepsy and for developing targeted therapeutic strategies. Despite the complexities, exploring the specific ways viruses trigger CNS alterations is crucial for developing early therapeutic interventions that target both the virus and inflammatory processes, potentially preventing the progression of epileptogenesis [56]. The clinical features of autoimmune and infectious status epilepticus differ, suggesting the need for tailored treatment approaches [57]. In the case of severe myoclonic epilepsy in infancy (SMEI), vaccination history and the risk of natural infection are important considerations, with the measles vaccine potentially increasing the risk of hyperthermia and convulsions [58]. In children with Dravet syndrome, the risk of seizures is significantly heightened by influenza infections. This was demonstrated in a study by Tro-Baumann et al., which found that seizures following vaccinations were common in these children, with the majority occurring after diphtheria, tetanus, and pertussis (DTaP) vaccinations and within 72 h [59]. Auvin et al. further highlighted the role of vaccinations in triggering seizures, with a proinflammatory immune response observed in children with Dravet syndrome [60]. Verbeek et al. and McIntosh et al. found that vaccination-associated seizure onset did not significantly alter the disease course but did not specifically address the impact of influenza infections on seizures [61,62]. It is important to note that while influenza infections may increase the risk of seizures in children with Dravet syndrome, the benefits of vaccination in preventing potentially life-threatening infectious diseases generally outweigh the risks of adverse events, including seizures. The risk of vaccination-associated seizures is vaccine-specific, with a low risk for certain vaccines. Therefore, healthcare providers need to engage in thorough discussions with patients and their caregivers, weighing the potential risks and benefits of each vaccine based on the individual’s medical history and the specific characteristics of the vaccines involved. This approach ensures that patients with Dravet syndrome receive the necessary protection from infectious diseases while minimizing the risk of adverse events, including seizures. As we navigate the challenges presented by the COVID-19 pandemic and potential future viral outbreaks, these insights into the intricate dynamics provide a foundation for understanding the mechanisms, developing targeted therapeutic strategies, and improving patient outcomes.

### 2.1. Mechanism Underlying Epileptogenesis and Neuroinflammation

There are different ways that viruses can reach the brain and infect the neural cells [63]. Upon penetrating the central nervous system, viruses can initiate an immunomodulatory response, constituting the host organism’s innate mechanism for combating infectious agents. However, sometimes, this immune response can be too strong or too prolonged and cause inflammation in the brain. Inflammation can damage the brain cells and alter their functions, such as communication, excitability, and plasticity. These changes can make the brain more prone to seizures and epilepsy [37,56].

These viruses can cause acute and delayed seizures through various mechanisms, including neuroinvasion, viral control and clearance, systemic inflammation, blood–brain barrier alterations, and inflammation-induced synaptic and neural circuit reorganization. The release of proinflammatory mediators by activated microglia and astrocytes can lead to a cascade of inflammatory processes in the brain (Figure 1), ultimately contributing to neuronal hyperexcitability and the development of seizures [64]. Microglia, the primary immune cells in the central nervous system, play a protective role in limiting virus distribution and persistence, thus preventing the development of seizures [65]. Infiltrating macrophages, on the other hand, are a key source of proinflammatory cytokines that can contribute to the development of seizures following viral infection [66]. Astrocytes, another type of glial cell, also play a significant role in the regulation of the immune response in epilepsy, with their activation of inflammatory pathways potentially contributing to seizure generation [67].

The release of proinflammatory cytokines (IL-1β, IL-6, and TNFα) can lead to neuroinflammation and disruption of the blood–brain barrier, which are associated with increased seizure susceptibility and the development of epilepsy [68]. Cytokines are small proteins that play a crucial role in intercellular communication and the regulation of immune responses. They can act on the cells that secrete them (autocrine action), on nearby cells (paracrine action), or, in some instances, on distant cells (endocrine action). Cytokines exert their effects by binding to specific cell surface receptors, leading to the activation of intracellular signaling pathways. This can result in the modulation of gene expression, cell proliferation, and the regulation of immune and inflammatory responses. Cytokines can stimulate their target cells to produce additional cytokines, leading to a cascade of immune and inflammatory responses. Some cytokines, such as IL-1β, can modulate the release of neurotransmitters in the central nervous system, affecting neuronal excitability and synaptic transmission. IL-6 and complement component 3 (C3) are cytokines especially implicated in seizure development. Distinct viral strains show varying pathways, with some relying more on cytokine activity and others on neuronal cell destruction [69]. For example, the replication of HIV in the CNS results in the stimulation of proinflammatory cytokines and neurotoxins, leading to oxidative stress. HIV can induce a cytokine storm in some patients, leading to neurological complications, such as seizures, encephalopathy, and dementia. Macrophages infected with the virus can form multinucleated giant cells, a classic feature seen in the pathology of HIV infection in the brain. Astrocytes act as a reservoir for the virus, which typically is dormant there unless the host cells make contact with lymphocytes or become activated directly by HIV gene products. IL-1β, IL-6, and TNFα are produced in response to HIV infection and contribute to the pathogenesis of neurological complications. They modulate neuronal excitability, disrupt neurotransmitter balance, and increase the permeability of the blood–brain barrier, all of which contribute to the generation and propagation of seizures [70,71]. These processes play a critical role in the coordination of immune and inflammatory responses, as well as the modulation of neuronal function and physiological homeostasis.

The resulting neuroinflammation can also lead to drug resistance in epilepsy, with unregulated inflammatory processes affecting the blood–brain barrier and neural network excitability [29]. Understanding these mechanisms is crucial for developing targeted therapeutic strategies, such as neuroimmunomodulation or anti-inflammatory therapy. Direct viral infection of neurons, as well as the indirect consequences of inflammation, hypoxia, edema, and oxidative stress, can lead to neuronal damage and death, disrupting the balance between inhibitory and excitatory neurons [72,73]. This imbalance can result in hyperexcitability and hypersynchrony of brain networks, potentially triggering seizures and epilepsy.

Another way that viruses can induce seizures and epilepsy is by binding to specific glutamate receptors on the surface of brain cells, such as *N*-methyl-D-aspartate (NMDA) receptors, which mediate excitatory neurotransmission and synaptic plasticity. This receptor is implicated in pre- and post-synaptic excitatory neurotransmission, leading to cellular and network hyperactivity and contributing to the formation of epileptogenesis [74,75]. This is further supported by the finding that seizures can elevate extracellular glutamate levels, which in turn can cause excitotoxic damage [76]. Additionally, exposure to glutamate can enhance the translation-dependent plasticity of group I metabotropic glutamate receptors, further promoting epileptogenesis [77]. By binding to these receptors, viruses can interfere with the normal functions of the cells and cause them to become overexcited or dysfunctional. For example, HSV-1 can bind to NMDA receptors on neurons and glial cells and cause neuronal hyperexcitability, synaptic dysfunction, and neuroinflammation [78].

A third way that viruses can induce seizures and epilepsy is by inducing changes in the brain’s structure and function, such as neuronal loss, neurogenesis (the formation of new neurons), gliosis (the proliferation of glial cells), and neural circuit alteration. These changes can affect the balance between excitation and inhibition in the brain, which is essential for normal brain activity. When this balance is disrupted, seizures and epilepsy can occur. HIV can induce neuronal loss, neurogenesis impairment, gliosis, and synaptic alterations in the brain, increasing the risk of seizures and epilepsy in infected patients [79,80]. In the case of COVID-19, its association with a range of neurological dysfunctions, including seizures and epilepsy, is thought to be due to the virus’s ability to bind to the angiotensin-converting enzyme 2 (ACE2) receptor, which is present in the brain and regulates blood pressure and inflammation [81]. The virus’s neuroinvasive and neurotropic capabilities and neuronal hyperexcitability are also believed to contribute to these neurological complications [82].

To better understand the mechanisms of viral-induced seizures and epilepsy, animal models are often used to mimic the human condition. One of the most widely used animal models is the mouse model of Theiler’s murine encephalomyelitis virus (TMEV) infection, which is part of the Picornaviridae family [83]. TMEV can induce transient encephalitic seizures in mice within a few days of infection, followed by a latent period of at least one week, and then spontaneous epileptic seizures in some mice, characterized by hippocampal sclerosis [84]. Stewart et al. demonstrated the onset of epilepsy in this mouse model, attributing it to potential molecular and cellular changes in glial and neural networks during the post-viral clearance latent period. However, the specific mechanisms driving epileptogenesis in this model remain unclear, prompting the need for further exploration [85]. Establishing a mouse model for viral-induced epilepsy holds significance for advancing epilepsy therapies, providing a valuable tool to understand the underlying mechanisms and assess innovative prevention and management strategies. However, while the mouse model enhances our understanding, acknowledging potential differences in viral-induced epilepsy mechanisms between mice and humans underscores the necessity for further investigations to comprehensively grasp this condition in humans. The precise mechanisms through which viruses impact the nervous system, ultimately leading to seizures and epilepsy, remain not entirely elucidated since these processes involve various factors, with a notable emphasis on neuroinflammation. Additionally, neuronal damage and death may contribute to the formation of glial scars, which can serve as a source of epileptogenicity, creating abnormal electrical activity or connections among neurons [86].

The brain’s ability to generate new neurons and form connections, known as neurogenesis and synaptic plasticity, is influenced by various factors, including injury and disease [87]. These processes hold promise for contributing to recovery from brain injury; however, it is crucial to exercise caution, as maladaptive changes may arise, potentially resulting in conditions such as epilepsy. Wojtowicz et al. further explored the impact of altered neurogenesis on learning and memory in the context of epilepsy [69], suggesting potentially negative effects. This study explored the potential consequences of altered neurogenesis on learning and memory in the epileptic brain. Plus, it discussed the historical connection between epilepsy and memory research, citing cases like patient H.M. [88] and autistic savant Daniel Tammet [89]. The authors delineated two theories concerning adult neurogenesis: the replacement theory, positing that new neurons replace dying ones, and the recruitment theory, suggesting that new neurons play an active role in input integration, thereby contributing to the process of learning. The authors conjectured that sudden and uncontrolled alterations in neurogenesis triggered by seizures may prove disruptive, potentially leading to cognitive impairments. The article concludes by cautioning that more neurogenesis is not always beneficial and that attempts to stimulate neuronal production for therapeutic purposes should consider proper integration within existing circuitry. At the same time, Di Filippo et al. proposed an immune-centered therapeutic approach to neurological disorders, emphasizing the role of inflammation in disrupting synaptic plasticity [90].

Further illustrating the mechanisms of epileptogenesis, the ability of HSV stands out due to its capacity to induce profound neuroinflammation. This involves the activation of microglia, astrocytes, and cytokines, along with the infiltration of leukocytes and antibodies. HSV can also cause neuronal damage and death, especially in the hippocampus, which is a key structure for memory and emotion. This widespread inflammation, edema, and parenchymal necrosis caused by HSV can lead to seizures and epilepsy, both during the acute phase of infection and as a late complication [91]. The neuropathology of HSV encephalitis in a rat seizure model has been studied, revealing site-specific CNS damage. The virus can also establish chronic latency in the brain, leading to long-term effects [92]. The role of the virus in immune evasion has been explored, with the virion host shutoff protein being identified as a potential mediator [93].

The rabies virus, a member of the Rhabdoviridae family, causes severe neuroinflammation and neuronal damage, particularly in the brainstem [94]. The virus is adept at evading the host’s immune response, allowing it to persist and spread within the nervous system. Due to the overexpression of immunosubversive molecules, like FasL, HLA-G, or B7-H1, in the infected neurological system, the rabies virus has evolved complex ways to kill or deactivate “protective” T cells that move into the infected brain system [95,96,97]. It can cause severe neuroinflammation, involving the activation of microglia, astrocytes, and cytokines, as well as the infiltration of leukocytes and antibodies [98,99]. Rabies virus can cause seizures and epilepsy, both during the acute phase of infection and as a late complication [94].

Finally, the COVID-19 virus exhibits various routes of entry into the brain, utilizing the olfactory nerve, bloodstream, or lymphatic system to infect critical regions, like the olfactory bulb, brainstem, cerebellum, thalamus, hypothalamus, and cortex [100]. This invasion triggers a spectrum of neuroinflammatory responses. Notably, the virus induces neuronal damage and death, particularly affecting the olfactory bulb, crucial for smell and taste. The neurological consequences include seizures and epilepsy, both in the acute phase of infection and as a late complication [101,102]. The neurological risks contribute to various complications, such as acute cerebrovascular accidents, acute necrotizing encephalopathy, and Guillain–Barré syndrome. These complications are more pronounced in severe COVID-19 cases and are associated with pre-existing conditions, like hypertension, diabetes, and chronic cardiac and respiratory diseases [103].

In summary, the mechanisms underlying the ability of viruses to induce seizures and epilepsy involve the induction of profound neuroinflammation, neuronal damage and death, and the establishment of chronic latency in the brain. These processes disrupt the normal functioning of the brain, leading to an increased risk of seizures and epilepsy in infected individuals.

### 2.2. Strategies for Disease Management

Treating seizures and subsequent epilepsy in patients with viral infections poses several challenges, such as the optimal timing, choice, dosage, and duration of anti-seizure medications. Some anticonvulsants may have adverse effects or interactions with antiviral drugs or other medications or may also affect the immune system and the inflammatory response, which may have implications for viral infection and epilepsy. The role of anti-inflammatory therapies, such as corticosteroids, monoclonal antibodies, and immunomodulators, in preventing or treating seizures and epilepsy secondary to viral infection is complex and multifaceted. These therapies may have beneficial or detrimental effects on the viral infection and epilepsy, depending on the type of virus, the stage of infection, the dose and duration of treatment, and the individual characteristics of the patient [104,105,106].

**Figure 3:**
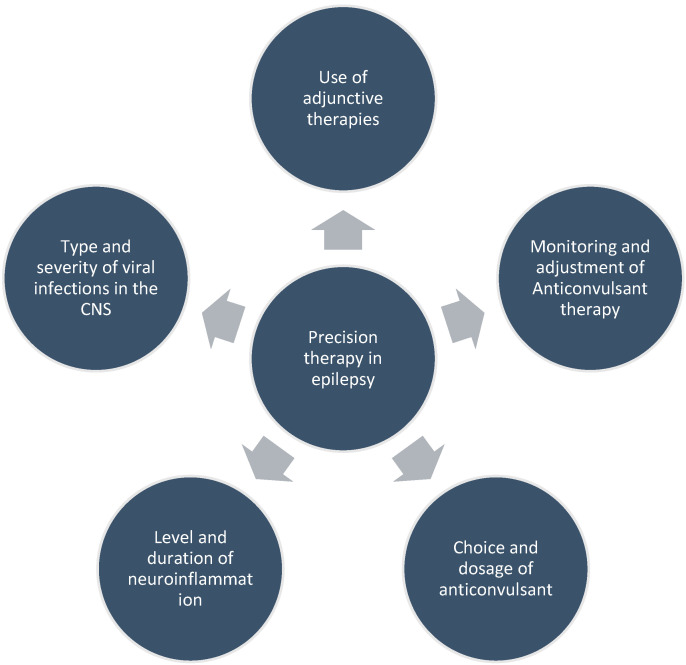
Aspects considered for the interplay of viral infection, neuroinflammation, and anticonvulsant drug pharmacokinetics in precision therapy in epilepsy.

Therefore, the management of epilepsy in individuals with viral infections requires a multidisciplinary approach, involving neurologists, infectious disease specialists, immunologists, pharmacologists, neurosurgeons, and other health professionals. To leverage the understanding of the interplay between viral infection, neuroinflammation, and treatment for precision therapies in epilepsy (Figure 3), it is important to consider several aspects, such as the following: (1) the type and severity of viral infection in the CNS, as different viruses may have different effects on the brain and the immune system; (2) the level and duration of neuroinflammation, as chronic or excessive inflammation may have detrimental effects on neuronal function and survival; (3) the choice and dosage of anticonvulsant, as they should be tailored to the individual patient’s characteristics, seizure type, and comorbidities; (4) the monitoring and adjustment of anticonvulsant therapy, as it may require frequent blood tests, dose titration, and switching of drugs to achieve optimal seizure control and minimize adverse effects; (5) the use of adjunctive therapies, such as anti-inflammatory agents, immunomodulators, or neuroprotective agents, that may have synergistic or complementary effects with anticonvulsants in reducing seizures and preventing epilepsy. Surgical treatments of epilepsy secondary to viral infection may not be effective or feasible in all cases, as the epileptogenic zone may be diffuse, multifocal, or deep-seated in the brain [107]. For example, some viruses may cause focal lesions, such as cerebral cavernous malformations (CCMs), that can be surgically removed with good seizure outcomes [108]. However, other viruses may cause diffuse or multifocal inflammation, such as in Japanese encephalitis or herpes simplex encephalitis, that may involve deep-seated structures, such as the hippocampus or the thalamus, which are difficult to access or resect without causing significant neurological deficits. The surgical outcome may also depend on the extent of brain damage, the presence of comorbidities, and the availability of resources [108,109].

## 3. Epilepsy Patients Acquiring Viral Infection

The underlying mechanisms of epilepsy are complex, involving a combination of genetic and acquired factors. While some cases are linked to known genetic mutations affecting ion channels and neurotransmitter receptors, others may result from brain injuries, strokes, tumors, infections, or congenital conditions [12]. Approximately 70% of seizures can be managed with medication [10]. Surgery, neurostimulation, or dietary modifications can then be taken into consideration for individuals whose seizures do not improve with medication (Figure 2). Many people with epilepsy get better to the point that they no longer require medication; therefore, not all occurrences of the condition are permanent [110]. The diagnosis involves ruling out other conditions that may mimic seizures, and confirmation often involves EEG testing, though a normal result does not rule out epilepsy.

Genetic alterations are believed to be significant contributors to the onset of specific epilepsy types. There are familial patterns in several epilepsies, suggesting the presence of an inherited gene or genes. On the other hand, de novo mutations, or spontaneous gene alterations, can cause epilepsy even in the absence of a family history. Researchers estimate the involvement of numerous genes, particularly in channelopathy-associated epilepsy, where variations in genes guiding ion channels, crucial for cellular ion flow regulation and neuronal signaling, are implicated. Beyond this, genetic changes affecting neuronal migration during brain development and those influencing carbohydrate breakdown are linked to epilepsy. While certain genetic changes may not directly cause epilepsy, they can impact susceptibility to seizures and responsiveness to anti-seizure medications. Genetic factors play a significant role, with certain mutations directly contributing to a small percentage of cases [111]. Most cases are thought to have some genetic component, either directly or indirectly. One or two percent of epilepsies are caused by a single-gene deficiency, while the majority are caused by the interaction of several genes with their environment [112]. There are almost 200 documented single-gene abnormalities, all of which are extremely unusual. The majority of implicated genes have direct or indirect effects on ion channels. These comprise the genes for GABA, enzymes, G protein-coupled receptors, and ion channels [112,113]. In identical twins, if one is affected, there is a 50–60% chance that the other will also be affected. In non-identical twins, the risk is 15%. These risks are greater in those with generalized rather than focal seizures. If both twins are affected, most of the time, they have the same epileptic syndrome (70–90%). Other close relatives of a person with epilepsy have a risk of five-times that of the general population [114,115].

Epilepsy may result from phakomatoses, a category of conditions caused by genetic abnormalities that affect the skin and central nervous system [116,117]. Compared to other phakomatoses, like neurofibromatosis type 1, certain phakomatoses, like tuberous sclerosis complex and Sturge–Weber syndrome, have a higher rate of epilepsy [118]. Tuberous sclerosis complex is an autosomal dominant disorder that is caused by mutations in either the TSC1 or TSC2 gene [119]. These mutations result in the upregulation of the mechanistic target of the rapamycin (mTOR) pathway. In addition, abnormal mTOR activity is believed to alter neural excitability. Relatively recent developments for the treatment of epilepsy in TSC patients include mTOR inhibitors, cannabidiol, and vigabatrin [120]. In the case of Sturge–Weber syndrome, the cause is an activating somatic mutation in the GNAQ. The mutation results in vascular malformations affecting the brain, skin, and eyes [121]. The prevalence of epilepsy is 75–100% and is higher in those with bilateral involvement. Seizures typically occur within the first two years of life and are refractory in nearly half of cases. However, high rates of seizure freedom with surgery have been reported in as many as 83% [122,123]. Neurofibromatosis type 1 is the most common phakomatose, and it is caused by autosomal dominant mutations in the Neurofibromin 1 gene. Clinical manifestations are variable but may include hyperpigmented skin marks, hamartomas of the iris called Lisch nodules, neurofibromas, optic pathway gliomas, and cognitive impairment [124,125].

In addition to these disorders, epilepsy can also arise from birth trauma, tumors, strokes, head trauma, prior CNS infections, genetic abnormalities, and brain damage sustained from birth [10]. About 35–70% of patients with brain tumors also have epilepsy, accounting for about 4 percent of cases [126]. Slow-growing tumors and those located in the temporal lobe provide the highest risk. Other mass lesions such as cerebral cavernous malformations and arteriovenous malformations have risks as high as 40–60%. Of those who have had a stroke, 6–10% develop epilepsy. Risk factors for post-stroke epilepsy include stroke severity, cortical involvement, hemorrhage, and early seizures. Between 6 and 20% of epilepsy is believed to be due to head trauma. Mild brain injury increases the risk about two-fold, while severe brain injury increases the risk seven-fold. In those who have experienced a high-powered gunshot wound to the head, the risk is about 50%. The risk of epilepsy following meningitis is less than 10%; it more commonly causes seizures during the infection itself [126,127].

### 3.1. Mechanisms Underlying the Development of Epilepsy

The mechanism of epilepsy involves disturbances in normal neuronal activity, potentially due to changes in ion channels, inhibitory neuron dysfunction, or alterations in excitatory and inhibitory circuit regulation. Understanding the mechanisms at different scales, from cellular to whole-brain levels, provides insight into the complexity of epilepsy [128]. During epilepsy, large numbers of neurons fire sequentially, resulting in non-synchronous electrical activity within the brain. Distinct elements within and outside the cell influence neuron activity, including variations in receptors, gene expression, and the kind, quantity, and location of ion channels. Ion concentrations, synaptic plasticity, and glial cell regulation further contribute to the factors surrounding the neuron [129]. The exact moment in which the brain’s excessive synchronization culminates in seizure activity is uncertain.

Voltage-sensitive ion channels are responsible for the depolarization of the nerve cell membrane and the conduction of action potentials across the surface of neuronal cells. The basic mechanisms of focal seizure initiation and propagation include high-frequency bursts of action potentials, the hypersynchronization of a neuronal population resulting in spike discharge on an EEG. Epileptiform activity at the single neuron level involves sustained depolarization, burst of action potentials, plateau-like depolarization, rapid repolarization, and hyperpolarization (paroxysmal depolarizing shift). Bursting activity is due to the Ca^2+^ influx, opening of voltage-dependent Na^+^ channels, Na^+^ influx, and generation of repetitive action potentials. Hyperpolarizing afterpotential is mediated by GABA receptors and Cl^−^ influx or K^+^ efflux, as well as the loss of surrounding inhibition and spread of seizure activity. Repetitive discharges lead to increased extracellular K^+^, accumulation of Ca^2+^ in presynaptic terminals, and NMDA receptor activation [129].

Spread occurs through local cortical connections and long association pathways like the corpus callosum. Anticonvulsants modulate these channels to reduce neuronal excitability. They also work to enhance gamma-aminobutyric acid (GABA)-mediated inhibitory neurotransmission and attenuate glutamate-mediated excitatory neurotransmission. An intriguing aspect suggests that immature astroglia, rather than immature neurons, may play a role in initiating brain activity leading to epileptic seizures.

Moreover, variations in the amounts of microRNAs (miRNAs) appear to be a major factor. MicroRNAs are a family of short non-coding RNAs that regulate the expression levels of several proteins by reducing the translation and stability of mRNA. As such, they may be important regulatory mechanisms and potential therapeutic targets in treating epilepsy [130,131]. In epilepsy, there is a reduced resistance of excitatory neurons to firing, which can lead to the formation of a specific seizure focus on the brain. Additionally, epilepsy may involve the overstimulation of excitatory circuits or the underactivity of inhibitory circuits following a brain injury, a process known as epileptogenesis. The breakdown of the blood–brain barrier may also contribute by allowing substances from the bloodstream to enter the brain [132,133].

As we have mentioned, inflammation within the CNS plays a pivotal role in epileptogenesis. Increased inflammatory states, marked by gliosis and microgliosis, are observed in the microenvironment of neural tissue. Pro-inflammatory cytokines are key players in modulating synaptic changes and neuronal hyperexcitability. These cytokines, along with other inflammatory mediators, contribute to the breakdown of the blood–brain barrier (BBB), allowing for leukocyte infiltration and exacerbating the inflammatory response. IL-1β, for instance, enhances glutamate release, promoting neuronal hyperexcitability, while TNFα regulates synapses and induces glutamergic transmission. IL-6, when upregulated, negatively impacts synaptic plasticity and neurogenesis. Prostaglandins, generated by astrocytes and microglia, further contribute to hyperexcitability and neuronal cell death. Additionally, chemokines, expressed in the brain, guide inflammatory mediators and activate leukocytes, playing a crucial role in epileptogenesis. The breakdown of the BBB, induced by inflammation, allows for leukocyte adhesion and infiltration, leading to the release of additional inflammatory mediators and exacerbating neurodegeneration. Understanding the neurobiology of inflammation in epileptogenesis is crucial for identifying biomarkers for patient screening and therapeutic targets for both preventing and treating epilepsy [132].

Epileptic seizures are not typically random events; they are often triggered by factors, such as stress, excessive alcohol consumption, flickering lights, or inadequate sleep. The seizure threshold, representing the stimulus level required for a seizure, is lowered in epilepsy. During a seizure, a group of neurons exhibits abnormal, excessive, and synchronized firing, resulting in a paroxysmal depolarizing shift. Normally, after an excitatory neuron fires, it becomes more resistant to firing due to inhibitory neurons, electrical changes within the neuron, and the negative effects of adenosine [128]. The emergence of focal and generalized seizures is influenced by a combination of network structure and excitability distribution [134]. This is further complicated by the fact that seizures can lead to structural and functional alterations in the brain [135]. In the context of focal epilepsy, atrophy in specific white matter tracts has been observed, but the exact cause of this atrophy is still unclear [136]. Additionally, the cellular mechanisms underlying generalized epilepsy may differ from those of focal epilepsy, with a potential role for inhibition in some forms of generalized epileptic discharges [137].

### 3.2. Understanding the Susceptibility of Epilepsy Patients to Viral Infections

The management of epilepsy primarily includes anticonvulsant medications, with the choice based on seizure type, syndrome, and individual factors. Surgery becomes a consideration for those who are medically refractory, aiming for total seizure control or significant reductions. Neurostimulation techniques, such as vagus nerve stimulation, offer alternative approaches for those ineligible for surgery (Figure 2). Beyond traditional treatments, emerging approaches like ketogenic diets and cannabidiol show promise in certain cases [138]. Psychological interventions and lifestyle adjustments, such as minimizing triggers, may complement medical treatments [139].

Epilepsy’s impact extends beyond the seizures themselves, influencing various aspects of an individual’s life. Individuals with epilepsy may be more susceptible to viral infections due to compromised immune function [140], drug interactions [141], and altered physiology [32]. For instance, patients with autoimmune epilepsy have a weakened immune system. These conditions may impair the ability of the immune system to fight off viral infections or increase the risk of autoimmune reactions that damage the brain or other organs [142]. Additionally, some anticonvulsants may have immunosuppressive effects, such as reducing the number or function of white blood cells, antibodies, or cytokines [143]. These effects may increase the susceptibility to viral infections or reduce the vaccine response.

Moreover, some anticonvulsants may interact with antiviral drugs, either by increasing or decreasing their blood levels or by causing adverse effects. For example, carbamazepine, phenytoin, or phenobarbital, may induce the activity of liver enzymes that metabolize antiviral drugs, such as acyclovir, valacyclovir, or oseltamivir, and reduce their effectiveness [144,145,146]. Conversely, some antiviral drugs, such as ritonavir, may inhibit the activity of liver enzymes that metabolize anticonvulsants, such as lamotrigine, and increase their toxicity [145,147]. In the context of epilepsy and viral infection, treatment with anticonvulsants and antiviral drugs should be carefully managed, with drug dosages adjusted as needed [148,149]. It is also important to consider the potential for interactions when prescribing anticonvulsants with other drugs, as they can induce hepatic enzymes and decrease the plasma concentration of many other medications [150,151]. New-generation anticonvulsants, such as levetiracetam, gabapentin, and pregabalin, are less likely to cause or be a target for clinically relevant pharmacokinetic drug interactions [152,153]. It has been established that oseltamivir, ganciclovir, and acyclovir may raise phenytoin plasma levels and result in toxicity [154]. The pharmacokinetics of anticonvulsants extend beyond considerations solely with antivirals; they also intersect with antibiotics and anti-inflammatory drugs. Phenytoin, carbamazepine, and lamotrigine may undergo metabolism influenced by certain antibiotics, including rifampicin, isoniazid, and erythromycin, leading to a reduction in their plasma levels [155,156,157]. Additionally, anti-inflammatory medications, such as ibuprofen, naproxen, and aspirin, can displace phenytoin and valproic acid from their protein binding sites, consequently increasing their free fractions [158,159,160]. These interactions have the potential to compromise seizure control, heighten adverse effects, or modify the pharmacokinetics of anticonvulsants.

Individuals with epilepsy may exhibit altered physiological conditions that influence their vulnerability to viral infections. These alterations encompass changes in parameters, like body temperature, blood pressure, heart rate, or respiratory function. For instance, certain individuals with epilepsy might have a subnormal body temperature, potentially compromising the immune response to viral infections and elevating the risk of hypothermia [161]. Conversely, others may present with an elevated body temperature, mimicking viral infection symptoms or heightening the risk of hyperthermia. Additionally, individuals with epilepsy who experience seizures impacting their breathing, such as apnea, hyperventilation, or aspiration, may face an increased susceptibility to respiratory infections. This vulnerability became particularly pronounced amid the COVID-19 pandemic [162,163]. There are some case studies or epidemiological evidence supporting the link between epilepsy and increased viral susceptibility. People with epilepsy who developed COVID-19 experienced seizure worsening and status epilepticus. The authors suggested that the viral infection, the cytokine storm, the medication interactions, and the physiological changes may have contributed to the seizure aggravation [164].

Similarly, individuals with epilepsy may experience physiological changes due to viral infections, including fever, dehydration, electrolyte imbalances, or acid–base disturbances. These changes can influence the seizure threshold, brain excitability, and the pharmacodynamics of anticonvulsant drugs. For example, fever may increase the neuronal firing rate, thereby reducing the seizure threshold, while dehydration could elevate the plasma concentration of anticonvulsants, leading to potential toxicity [165]. Electrolyte imbalances, such as hyponatremia, might induce cerebral edema and raise intracranial pressure. Acid–base disturbances, like acidosis, can alter the ionization and distribution of anticonvulsants, impacting their binding and transport. These alterations may contribute to seizure exacerbations, status epilepticus, or neurological complications [166,167,168]. The COVID-19 pandemic heightened anxiety, depression, and somnolence in individuals with epilepsy, potentially exacerbating their condition [169]. Children with a severe form of epilepsy should be vaccinated against the flu due to the high risk of seizures being triggered by an influenza infection. The safe administration of the seasonal influenza vaccine should be a priority in those with SCN1A-positive Dravet syndrome, given the likelihood of severe neurological symptoms and complications, such as worsening seizures, deteriorating language and motor skills, and even death after catching the flu [170,171].

## 4. Pharmacokinetic Considerations in Epilepsy and Viral Infection

Viral infections can profoundly impact the pharmacokinetics of anticonvulsants, influencing both their efficacy and safety profiles. This can lead to instances of subtherapeutic or supratherapeutic drug levels, consequently diminishing efficacy or elevating the risk of toxicity. Hence, it becomes crucial to closely monitor drug levels, viral load, and clinical responses in epilepsy patients with viral infections. Adjusting the dose or selecting anticonvulsants should be carried out carefully, especially in cases where polytherapy to manage epilepsy is necessary. The complexity increases when other diseases are involved, demanding a thorough assessment of potential drug interactions. Notably, drug metabolism, primarily mediated by enzymes like those in the liver’s cytochrome P450 (CYP) system, can be influenced by viral infections. These infections may induce or inhibit these enzymes, consequently impacting the rate and extent of drug metabolism [172]. Viral infections can affect the absorption/bioavailability of drugs, either by altering the physiological conditions of the gut or by causing symptoms, such as vomiting, diarrhea, or loss of appetite, which can reduce the intake or retention of the drug. Phenytoin, carbamazepine, and levetiracetam solubility characteristics can impact their absorption and potential interactions with bile secretion or composition. According to the Biopharmaceutics Classification System (BCS), phenytoin and carbamazepine are classified as having low solubility (Class II), while levetiracetam is classified as having high solubility (Class I). Changes in bile secretion or composition, which can occur during viral infections or due to the use of antiviral drugs, have the potential to affect the absorption of medications that are sensitive to such changes. Additionally, valproic acid, gabapentin, and pregabalin have pH-dependent absorption, which can be affected by changes in gastric acidity caused by viral infections or antiviral drugs [173]. Viral infections can affect the clearance of drugs, either by impairing the function of the organs involved in drug elimination or by causing inflammation or edema that can reduce the blood flow to these organs [174]. Phenytoin, valproic acid, and lamotrigine are mainly cleared by the liver. These medications can be affected by viral infections or antiviral drugs that cause hepatotoxicity or cholestasis. For example, hepatic biotransformation accounts for 70% clearance of valproic acid, and the rest is excreted unchanged by the kidneys [175]. Levetiracetam, topiramate, and zonisamide are mainly cleared by the kidneys [176]. These medications can be affected by viral infections or antiviral drugs that cause nephrotoxicity or renal impairment. For instance, renal or hepatic disease can prolong the elimination of the parent drug or an active metabolite, leading to accumulation and clinical toxicity [150].

Pharmacokinetics can also be affected by neuroinflammation. During neuroinflammatory disorders, the BBB may be compromised, leading to changes in drug delivery to the brain. This can result in both increased and decreased drug permeability, affecting the efficacy of anticonvulsants [177,178,179]. There is a need for anticonvulsants with limited pharmacokinetic interactions, suitable dosing preparations, and parenteral formulations, which are particularly important in the context of neuroinflammation [34]. Anticonvulsants work by modulating the activity of different neurotransmitters, such as glutamate, GABA, and sodium and calcium channels [152]. Antivirals are drugs that are used to inhibit the replication or spread of viruses by interfering with different stages of the viral life cycle, such as entry, uncoating, transcription, translation, assembly, or release [180]. Because neuroinflammation can modify the expression and functionality of the enzymes, like cytochrome P450, that metabolize these medications, the levels and efficacy of the pharmaceuticals can be changed. Reduced drug absorption and availability in the brain can result from neuroinflammation’s impact on the movement and dispersion of these drugs across the blood–brain barrier. Moreover, neuroinflammation can modulate the activity and sensitivity of the neurotransmitter systems that are targeted by anticonvulsants, resulting in reduced or enhanced anticonvulsant effects. Moreover, it can influence the immune response and the viral load in the brain, affecting the antiviral effects and the risk of viral reactivation [181,182,183,184].

### 4.1. Drug Interactions

While not all antiviral–anticonvulsant drug interactions are clinically significant, there is a need to be cautious about potential interactions between these drug classes. Some anticonvulsants and antivirals can have additive, synergistic, or antagonistic effects on each other, either pharmacodynamically (by affecting the same or different targets) or pharmacokinetically (by affecting the absorption, distribution, metabolism, or excretion of each other). Examples of antiviral drugs and anticonvulsants that have been reported to have potential interactions include Atazanavir and remdesivir, which have potential interactions with anticonvulsants, such as cannabidiol (CBD) [185]. These interactions can lead to decreased concentrations of some medications or their compositions, which may affect their efficacy. Valproic acid has been reported to have potential interactions with antiviral drugs like darunavir/cobicistat and lopinavir/ritonavir. These interactions can also lead to decreased concentrations of some medications or their compositions, which may affect their efficacy [34,35,145,155].

Despite the potential for significant interactions between anticonvulsants and antivirals, there are cases where these interactions have no clinical significance [33]. The first-generation anticonvulsants, including phenytoin, carbamazepine, and phenobarbital, are potent inducers of CYP450 isoenzymes and drug transporters. Due to their potential to compromise direct-acting antiviral (DAA) serum concentrations and elevate the risk of treatment failure and resistance, co-administration with DAAs is currently not recommended. This presents a challenge for patients on first-generation anticonvulsants for seizure disorders and initiating hepatitis C virus (HCV) treatment with standard-dose DAAs in these individuals necessitates a meticulous, patient-specific evaluation, weighing the risks against the benefits [186]. The challenges of administering DAAs for hepatitis C virus and first-generation anticonvulsants include potential drug–drug interactions, suboptimal drug levels, and treatment failure. These challenges can lead to suboptimal seizure control and adverse effects in patients with epilepsy and hepatitis C infection. Therefore, the co-administration of DAAs and first-generation anticonvulsants is not currently recommended due to the potential for drug–drug interactions, which can result in significantly reduced DAAs levels and the risk of treatment failure. However, some case reports have described successful outcomes in patients receiving HCV DAA treatment while remaining on first-generation anticonvulsants [186]. Patients who are unable to stop or switch to alternative anticonvulsant agents may face challenges in adhering to HCV treatment. There is limited clinical experience with the co-administration of DAAs and first-generation anticonvulsants, which may lead to hesitancy in prescribing HCV DAA treatment in this patient cohort [186].

Second-generation anticonvulsants or alternative dose/time regimens may provide better treatment outcomes and reduce the risk of drug interactions when treating patients with HIV infection and seizure disorders. The co-administration of DAAs with anti-epileptics or mood stabilizers with cytochrome P450 inducers can be managed using pharmacokinetic enhancers [187]. These enhancers can help minimize drug–drug interactions and improve the effectiveness of HCV treatment. The choice of specific DAA combinations may depend on the patient’s HCV genotype, presence of cirrhosis, and other factors. For example, in a case report involving a patient with HCV genotype 1a, the recommended treatment was glecaprevir/pibrentasvir, but the concomitant use of phenytoin and glecaprevir/pibrentasvir was not recommended due to a drug interaction [188]. Alternative DAA combinations may need to be considered in such cases. Individualized dose adjustments may be necessary to minimize drug–drug interactions and achieve optimal treatment outcomes.

In cases where first-generation anticonvulsants cannot be adjusted or switched, the use of second-generation anticonvulsants may provide better treatment outcomes and reduce the risk of drug interactions. Second-generation anticonvulsants, such as levetiracetam, lamotrigine, gabapentin, and brivaracetam, may be more favorable options for patients on antiviral treatment, as they have less of an effect on the enzymatic function of CYP3A4 [3,188]. These anticonvulsants may be less likely to interfere with antiviral drug concentrations or cause significant drug–drug interactions. For instance, lamotrigine and efavirenz can be safely co-administered without dose adjustment or increased monitoring. According to the HIV guidelines from Clinical Info HIV and the American Academy of Neurology, co-administration of these two drugs does not require a lamotrigine dosage adjustment. However, it is essential to monitor the patient’s seizure control and plasma concentrations of lamotrigine. Additionally, the co-administration of raltegravir and lamotrigine may not require a lamotrigine dosage adjustment [189,190].

Drug interactions are complex phenomena that depend on many factors, such as the pharmacokinetics, pharmacodynamics, pharmacogenetics, and pharmacovigilance of the drugs involved. Measuring the correct outcomes is essential to assess the clinical relevance of drug interactions, but it is not always easy or feasible. The following may lead to an underestimation of their prevalence and impact: the lack of standardized definitions and methods for identifying, classifying, and reporting drug interactions; the variability in the severity and frequency of adverse reactions resulting from drug interactions, which may depend on individual patient characteristics, co-morbidities, concomitant medications, and environmental factors; the difficulty in establishing a causal relationship between a drug interaction and an adverse outcome, especially when multiple factors are involved or when the outcome is delayed or rare; the underreporting and underrecognition of drug interactions in clinical practice and research. Therefore, it is possible that some drug interactions may have clinically relevant outcomes that are not measured or detected by the current methods and tools. This may result in missed opportunities for preventing or managing potential harm to patients. Establishing clear and standardized criteria and guidelines is imperative for identifying, evaluating, and reporting drug interactions and their outcomes. The integration of advanced analytical and statistical methods, including artificial intelligence, machine learning, and network analysis, plays a crucial role in discerning and predicting drug interactions and outcomes. Additionally, these methods facilitate the generation of personalized, evidence-based recommendations to inform clinical decision making [191,192]. However, not all drug interactions are clinically significant, and some may be beneficial or negligible. The clinical significance of drug interactions depends on various factors, such as the dose, timing, route, and duration of administration of the drugs, the pharmacological properties and mechanisms of the drugs, the genetic and physiological characteristics of the patient, and the presence of other diseases or conditions that may affect the drug response. Drug interactions are especially important to consider, as both conditions may require multiple medications that can interact with each other.

Recent viral outbreaks, such as COVID-19, have posed significant challenges and opportunities for the management of neuroinflammation and the use of anticonvulsants and antivirals. COVID-19 patients may require anticonvulsants and antivirals, either as prophylaxis or treatment, depending on their clinical situation. However, the optimal choice, dose, and duration of these drugs are not well established, and the potential interactions and adverse effects of these drugs are not well known and may vary depending on the individual patient characteristics, such as age, gender, comorbidities, and genetic factors. Therefore, COVID-19 patients needing anticonvulsants and antivirals should be carefully monitored and their drug regimens adjusted accordingly [193]. A table describing certain interactions between these two classes of medications was made available from the Italian League Against Epilepsy (https://www.lice.it/pdf/Anticonvulsant_drugs_interactions_in_COVID-19.pdf, accessed on 7 December 2023). Plus, the Liverpool Drug Interaction Group, in collaboration with other institutions, maintains an evolving list of clinically relevant drug–drug interactions between anticonvulsant drugs and medications used in treating COVID-19 patients. The table outlines interactions for various drugs, including antivirals (e.g., atazanavir, remdesivir), chloroquine, and tocilizumab. The interactions are categorized as potential increases or decreases in exposure or as no significant effect. For instance, anticonvulsants, like carbamazepine, ethosuximide, and valproic acid, may show varying interactions with COVID-19 drugs. Importantly, single-case management is emphasized due to the pharmacological complexity, and the table provides a comprehensive overview for medical practitioners. The notes highlight specific considerations, such as the lack of evidence for darunavir-based treatments for SARS-CoV-2 and the absence of certain data for remdesivir interactions. Additionally, potential effects on plasmatic concentrations are mentioned, emphasizing the need for caution and individualized approaches in managing drug interactions during the treatment of COVID-19 in patients with epilepsy. (https://www.covid19-druginteractions.org/, accessed on 7 December 2023). Levetiracetam is one of the medications under investigation that is very intriguing because it interacts with COVID-19 medications very little. This may make it more appropriate as a recently added anti-seizure drug to relieve seizures following infection with SARS-CoV-2 [194,195].

### 4.2. Treatment of Individuals with Epilepsy Who Acquired a Viral Infection Versus Those with Virus-Induced Epilepsy

The treatment of individuals with epilepsy who acquired a viral infection and those with virus-induced epilepsy may have some similarities and differences, depending on the type and severity of the infection, the type and frequency of the seizures, and the response and tolerance to the anticonvulsants and antivirals. Both groups of patients may require anticonvulsants to prevent or treat seizures, especially if they have a history of epilepsy or other risk factors. The choice of anticonvulsants should be based on the seizure type, the efficacy, the safety, and the potential interactions with antivirals or other medications. Individuals with virus-induced epilepsy may have a persistent or progressive increase in seizure frequency or severity after the acute phase of infection, which may persist or worsen over time. They may need to adjust their anticonvulsant regimen, either by adding new drugs, increasing the dose, or switching to different drugs, to achieve better seizure control. They may also need to continue the antivirals indefinitely, or until the viral infection is cleared or suppressed, to prevent viral reactivation or recurrence.

Patients in both groups stand to gain from antiviral therapy, which can impede viral replication and diminish the viral load in the brain. This, in turn, holds promise for alleviating neuroinflammation and reducing susceptibility to seizures. The selection of antiviral agents should be guided by factors, like the specific virus involved, efficacy, safety profile, and potential interactions with anticonvulsants or other medications. Monitoring the blood levels of both anticonvulsants and antivirals, along with regular assessments of liver and kidney functions, is imperative for both patient cohorts. This vigilant oversight ensures optimal dosage, averting the risk of toxicity or adverse effects. Adhering to general recommendations for epilepsy management is equally crucial for both sets of patients. This encompasses steering clear of triggers, maintaining a consistent sleep routine, prioritizing hydration, and effectively managing stressors. This comprehensive approach contributes to a holistic and effective care strategy for individuals dealing with epilepsy and viral infections. However, individuals with epilepsy who acquired a viral infection may have a transient increase in seizure frequency or severity during the acute phase of infection, which may subside after recovery. They may not need to change their anticonvulsant regimen, unless their seizures are poorly controlled or their drug levels are altered by the infection or the antivirals. They may also not need to continue the antivirals after the infection is resolved unless they have a chronic or recurrent viral infection [32,196].

Therefore, understanding mechanistic drug–drug interactions (DDIs) is paramount in optimizing treatment outcomes. Mechanistic DDIs can be further classified into pharmacokinetic or pharmacodynamic DDIs. Pharmacokinetic DDIs involve changes in the absorption, distribution, metabolism, or excretion of one or both drugs, while pharmacodynamic DDIs involve changes in the efficacy or toxicity of one or both drugs. Mechanistic DDIs can be predicted using in vitro data, in silico models, or clinical studies. Static DDIs are based on the magnitude of the change in the exposure of one drug when co-administered with another drug. Static DDIs are usually expressed as the ratio of the area under the plasma concentration–time curve (AUC) or the peak plasma concentration (Cmax) of the drug in the presence and absence of the interacting drug. Static DDIs can be predicted using mechanistic static models or physiologically based pharmacokinetic (PBPK) models. The difference between mechanistic and static DDIs explains why some drugs may show an interaction in silico but not in vivo. This is because in silico models may not capture all the factors that influence the DDI outcome in vivo, such as the variability in drug concentrations, the interplay of multiple enzymes and transporters, the effect of food or other co-medications, the genetic polymorphisms, the disease state, or the adaptive responses of the body. Therefore, in silico models should be validated with clinical data whenever possible, and the limitations and uncertainties of the models should be acknowledged [197,198].

### 4.3. Modeling of Pharmacokinetics

PBPK modeling in predicting the pharmacokinetics of anticonvulsants during viral infections can provide mechanistic and quantitative insights into the complex interactions between the drug, the virus, and the host. It can reduce the need for costly and time-consuming clinical trials, especially in situations where patient recruitment is challenging or unethical. It can support the development of personalized medicine and precision dosing by accounting for the variability and uncertainty in the individual factors that influence the drug response [199]. PBPK modeling can be used to simulate the pharmacokinetics of drugs in different scenarios, such as drug–drug interactions, special populations, alternative dosing regimens, and disease states. PBPK modeling can be used to estimate the impact of viral infections on the exposure and response to anticonvulsants and to inform personalized dosing strategies to optimize treatment outcomes [200]. In Stader et al., a PBPK model was created to examine how the COVID-19 cytokine storm affected the pharmacokinetics of concurrently administered medications, such as midazolam, which is metabolized by CYP3A enzymes. This allowed for the efficient study of clinical scenarios and the management of novel disease treatments [201].

PK and PBPK modeling also has some limitations, such as the reliability and quality of the input data, the validation and verification of the model assumptions and parameters, and the evaluation of the model performance and sensitivity. It relies on the availability and quality of the input data, such as the drug properties, the system parameters, and the infection-related changes. The data may be scarce, incomplete, inconsistent, or uncertain, which may affect the accuracy and reliability of the model predictions. Moreover, it requires the validation and verification of the model assumptions, structure, and parameters, as well as the evaluation of the model performance and sensitivity. The validation process may be challenging, especially when there is a lack of clinical data or gold standard for comparison. It may not capture all the relevant factors and mechanisms that influence the pharmacokinetics and pharmacodynamics of anticonvulsants during viral infections, such as the immune response, the viral load, the disease severity, and the co-administration of other drugs. The model may need to be updated and refined as new knowledge and data become available. Despite these limitations, PK and PBPK modeling can reduce the need for costly and time-consuming clinical trials, especially in situations where patient recruitment is challenging or unethical [202,203].

PK and PBPK modeling plays a pivotal role in the early stages of antiviral drug development, offering invaluable insights into predicting the pharmacokinetics and pharmacodynamics of candidate drugs across diverse human populations. This is exemplified by studies on remdesivir, an FDA-approved drug for COVID-19 treatment. The whole-body PBPK modeling illustrated how remdesivir’s metabolites exhibit varied exposure in different tissues, emphasizing the importance of understanding tissue-specific pharmacokinetics. PK and PBPK modeling also proves crucial in designing and optimizing dosing regimens, not only for antiviral efficacy but also for preventing comorbidities like epilepsy in susceptible populations, particularly those with viral infections in the central nervous system. These modeling approaches enable a more personalized and effective approach to drug administration. Moreover, in the context of viral outbreaks, PK and PBPK models offer insights into potential drug interactions, aiding in the strategic management of diseases. By simulating drug exposure and metabolism in various scenarios, these models empower healthcare professionals to make informed decisions regarding treatment protocols, ensuring optimal therapeutic outcomes. The integration of PK and PBPK modeling in antiviral drug development proves instrumental in predicting drug behavior, optimizing dosing strategies, and enhancing our understanding of potential complications and interactions, ultimately contributing to more effective and tailored therapeutic interventions [204,205].

## 5. Interactions at the Neuroinflammatory Level

Viral infections that cause epilepsy and those with epilepsy who either subsequently contract a virus or are more likely to do so because of fragilities brought on by epilepsy are intricately linked. Infections contribute to the progression of epilepsy through inflammation, and early-onset seizures brought on by viral infections (especially encephalitis) are thought to be a risk factor for the development of chronic epilepsy in the future [56]. Viral infections can lead to early seizures as an acute consequence of the infection, while epilepsy patients may acquire a viral infection later, which can potentially affect the severity and frequency of seizures. Both viral infections inducing epilepsy and epilepsy patients acquiring a viral infection share similarities in terms of their impact on seizures, epileptogenesis, and inflammation. It is important to note that the timing and specific pathophysiological mechanisms may differ between the two scenarios.

Neuroinflammation can have beneficial and detrimental effects on the brain, depending on the stimulus type, intensity, and duration, and epileptogenesis is a hypothetical process by which brain networks are transformed to generate unprovoked seizures and consists of three periods: (1) the initial insult; (2) the latent period; and (3) the chronic phase. The initial seizure onset can be a result of idiopathic causes or previous neurological insults, such as de novo status epilepticus, traumatic brain injury, brain tumor, infection, and ischemia. The initial phase is followed by a latent period involving the development of maladaptive neuroinflammation without apparent seizure activity. The latent period can last for days, weeks, or months and then progresses into the chronic phase, which is characterized by spontaneous recurrent seizures along with sustained neuroinflammation [206].

### 5.1. Molecular and Cellular Mechanisms of Neuroinflammation

Neuroinflammation is a process that involves the activation of immune cells and the release of inflammatory mediators in the brain. Therefore, neuroinflammation can be both a cause and a consequence of epilepsy. Neuroinflammation can play a role in the origin and development of epilepsy by affecting the excitability, transmission, and plasticity of neurons and synapses, and by altering the balance between excitation and inhibition in the brain [207]. Neuroinflammation can be different between patients with epilepsy and patients with viral infections that develop epilepsy, depending on the type and severity of the viral infection, the time and duration of the infection, and the individual susceptibility and response to the infection. Understanding the neurobiology of neuroinflammation in epilepsy can help to identify and validate new biomarkers and therapeutic targets for the diagnosis, prognosis, and treatment of epilepsy. For example, neuroinflammation may play a more prominent role in the early phases of epileptogenesis than in the chronic stages of epilepsy, when the brain may develop compensatory or protective mechanisms. Moreover, neuroinflammation may have different effects on distinct types of epilepsy, such as focal or generalized, or on different seizure subtypes, such as tonic–clonic or absence [27]. Neuroinflammation is a mediator in epileptogenesis following viral infections, and it can influence the pathophysiology and clinical manifestations of epilepsy. Neuroinflammation can interact with epilepsy in a bidirectional and multifaceted manner, and it may offer new opportunities for diagnosis and treatment. However, more research is needed to elucidate the specific mechanisms and effects of neuroinflammation in different forms and stages of epilepsy and to optimize the safety and efficacy of anti-inflammatory interventions.

The intricate relationship between genetics, neuroinflammation, and epilepsy is exemplified in certain genetic forms of epilepsy that impact the expression or function of molecules involved in the inflammatory response within the brain. This intricate interplay can lead to distinct clinical manifestations [208]. For example, mutations in the gene encoding for interleukin-1 receptor antagonist (IL1RN) can cause a rare form of epilepsy called deficiency of IL1RN, which is characterized by recurrent febrile seizures, systemic inflammation, and skin lesions [209]. Similarly, mutations in the gene encoding for toll-like receptor 3 (TLR3) can cause another rare form of epilepsy, which is characterized by severe brain inflammation and seizures triggered by herpes simplex virus infection [210]. These mutations have been found to increase susceptibility to herpes simplex virus encephalitis, a serious complication of HSV and varicella-zoster virus (VZV) infections. These examples highlight how genetic alterations can impact crucial components of the inflammatory response, contributing to the development of epilepsy with distinct clinical phenotypes.

Acute neuroinflammation caused by head trauma or infections is linked to some acquired forms of epilepsy, such as post-traumatic or post-infectious epilepsy. This inflammation contributes to increased glutamate release, the primary excitatory neurotransmitter, and decreased GABA release, the primary inhibitory neurotransmitter, potentially leading to seizures during the acute phase [24]. For instance, cytokines like TNFα or IL-1β can enhance sodium and calcium channel expression and function, increasing ion influx, depolarization, and firing [211]. Additionally, pro-inflammatory cytokines can boost the expression and function of glutamate receptors, such as *N*-methyl-D-aspartate (NMDA) or alpha-amino-3-hydroxy-5-methyl-4-isoxazolepropionic acid (AMPA) receptors, resulting in elevated sodium and calcium influx, leading to excitotoxicity and neuronal damage [211,212]. Simultaneously, these cytokines may diminish the expression and function of GABA receptors, like GABA-A or GABA-B receptors, reducing chloride influx, causing hyperpolarization, and promoting inhibition [213].

Neuroinflammation can also contribute to the development of epilepsy in the chronic phase by inducing structural and functional changes in the brain that lead to the formation of an epileptogenic focus, a region of the brain that is prone to generating spontaneous seizures [24]. For example, chronic activation of microglia, the resident immune cells of the brain, can release pro-inflammatory cytokines, chemokines, and reactive oxygen species, which can cause neuronal death, astrocyte activation, blood–brain barrier disruption, and vascular remodeling. Chronic activation of astrocytes can cause neuronal damage, microglial activation, synaptic pruning, and aberrant sprouting [214,215].

Potential therapeutic targets for reducing neuroinflammation in acquired epilepsy include molecules and pathways involved in neuroinflammatory processes, such as microglial activation, reactive astrogliosis, disruption of the blood–brain barrier (BBB), inflammatory cellular infiltration, neuronal loss, neuronal plasticity, and circuit reorganization. However, the specific targets and strategies for reducing neuroinflammation in acquired epilepsy are still being investigated. Neuroinflammation can protect the brain from infection by eliminating the pathogens and repairing the damage, but it can also cause collateral damage to healthy brain cells and disrupt the normal functioning of the neurons and synapses. Neuroinflammation can also alter the blood–brain barrier, the protective layer preventing harmful substances and immune cells from entering the brain. The interplay between viral infections, neuroinflammation, and epilepsy is a complex and dynamic phenomenon that involves multiple factors and mechanisms (Figure 1). Viral infections can cause epilepsy by infecting the brain and triggering inflammation, damage, or abnormal electrical activity. Epilepsy patients can also acquire viral infections due to their impaired immune system, anticonvulsant drugs, or surgical interventions. Neuroinflammation is a common feature of both scenarios, but it may differ in its onset, duration, intensity, and consequences. In case of the herpes simplex virus, it can cause encephalitis and induce seizures and epilepsy, both during the acute phase of infection and as a late complication. Rabies virus can also cause encephalitis and induce seizures and epilepsy but usually with a longer latency period. COVID-19 virus can cause mild to severe neuroinflammation and induce seizures and epilepsy, both during the acute phase of infection and as a late complication [216,217,218].

In epilepsy patients acquiring infections, neuroinflammation is usually a chronic and persistent condition that results from the underlying cause of epilepsy. Neuroinflammation can also be exacerbated by anticonvulsant drugs, surgical interventions, or the comorbidities associated with epilepsy. Neuroinflammation can, therefore, affect the pharmacokinetics of anticonvulsant drugs, the immune system’s function, the brain’s physiology, increase the vulnerability to viral infections, and reduce vaccine response. For example, neurocysticercosis (preventable parasitic infection caused by larval cysts of the pork tapeworm), despite causing seizures and epilepsy, can also increase the prevalence of viral infections, such as hepatitis B, hepatitis C, and HIV, due to shared risk factors, such as poor sanitation, low socioeconomic status, or lack of access to health care [219]. The host’s immune response to the cysts, rather than the cysts themselves, is responsible for the symptoms and complications of neurocysticercosis. The immune response can lead to inflammation, edema, and the development of granulomas around the cysts, which can result in seizures, headaches, and other neurological symptoms. The severity of the symptoms largely depends on the location and number of cysts, as well as the host’s immune response. The immune response to the cysts is typically more localized and focal, and it can be chronic and persistent, as the cysts can persist in the CNS for extended periods, leading to ongoing immune activation and inflammation [220]. On the other hand, HIV-associated neuroinflammation is a result of the direct effect of the virus on the peripheral and central nervous system. The neuroinflammation associated with HIV is more diffuse and widespread, as it is a response to the presence and long-term infection of the virus in the CNS. Both can be chronic and persistent, but the nature and extent of the neuroinflammation differ based on the underlying cause. Both can be chronic and persistent, but the underlying causes and the nature of the immune response are distinct.

Different viruses may have different routes of neuroinvasion, replication, and clearance, and they may induce different types and degrees of neuroinflammation [32]. Seizures can result directly from the invasion of the virus into the CNS or indirectly through the immune responses mounted by the host. Herpes simplex virus [221,222], influenza virus [223,224,225,226], or SARS-CoV-2 [227] can directly infect the brain and cause encephalitis, while other viruses, such as measles virus [228,229] or mumps virus [230,231,232], can indirectly affect the brain and cause encephalopathy. Finally, HIV [233,234,235], cytomegalovirus, or Epstein–Barr virus [236], can persistently infect the brain and cause chronic neuroinflammation and seizures. The timing and duration of the viral infection can affect the onset and progression of epilepsy. Herpes simplex virus [221], influenza virus [226], or SARS-CoV-2 [227] can cause acute seizures during or shortly after the infection, which may or may not evolve into chronic epilepsy. Other viral infections, such as measles virus [229] or mumps virus [231], can cause delayed seizures months or years after the infection, which may or may not be associated with chronic epilepsy. Some viral infections, such as human immunodeficiency virus [234,237], cytomegalovirus [238], or Epstein–Barr virus [239,240], can cause recurrent seizures throughout the infection, which may or may not be controlled by antiviral or anti-epileptic drugs.

The individual susceptibility and response to the viral infection can affect the risk and outcome of epilepsy. Environmental factors, such as age, sex, comorbidities, or medication, can also modulate the inflammatory response to the viral infection and the susceptibility to epilepsy [167]. Some immunological factors, such as the type and magnitude of the immune response, the balance between pro-inflammatory and anti-inflammatory pathways, and the resolution and repair of the inflammation, can also modulate the inflammatory response to the viral infection and the outcome of epilepsy [241].

In some cases, neuroinflammation can exacerbate seizures and epilepsy by increasing neuronal hyperexcitability and synaptic transmission, and by inducing neuronal damage and death. In other cases, neuroinflammation can attenuate seizures and epilepsy by enhancing neuronal inhibition and neuroprotection, and by facilitating the repair and regeneration of the nervous tissue. The outcome of neuroinflammation in epilepsy may depend on the balance between the pro-inflammatory and anti-inflammatory mediators, and the interaction between the innate and adaptive immune systems [207].

The role of neuroinflammation in epilepsy with viral infections may differ from the role of neuroinflammation in epilepsy in scenarios with no viral infections since viral infections can trigger specific immune responses that are not present in other causes of epilepsy. For example, viral infections can activate the TLRs and the retinoic acid-inducible gene I (RIG-I)-like receptors (RLRs), which are pattern recognition receptors that recognize viral nucleic acids and initiate the production of type I interferons (IFNs) and other cytokines [242,243,244]. Type I IFNs can have antiviral and immunomodulatory effects but they can also affect neuronal function and viability and modulate the expression of anticonvulsant drug transporters and receptors [245]. Viral infections can also induce the production of autoantibodies that can cross-react with neuronal antigens and cause autoimmune encephalitis, which is a condition characterized by inflammation of the brain, which can lead to a variety of neurological symptoms, including seizures, and is often refractory to conventional anticonvulsants [246,247,248]. Understanding these mechanisms can help to identify novel biomarkers and therapeutic targets for epilepsy. More studies are needed to elucidate the precise role of neuroinflammation in epileptogenesis and to evaluate the safety and efficacy of anti-inflammatory therapies in epilepsy.

### 5.2. Identification of Biomarkers

The quest for biomarkers associated with neuroinflammation that could serve as early indicators of epilepsy risk is a challenging but promising research area. Several putative molecular, imaging, electroencephalographic, and behavioral biomarkers of epileptogenesis have been identified, but clinical translation has been hindered by fragmented and poorly coordinated efforts, issues with inter-model reproducibility, study design, statistical approaches, and difficulties with validation in patients.

Specific molecular alterations in plasma or serum could reflect alterations in brain function associated with epileptogenesis, such as dendritic remodeling, neuronal hyperexcitability, axonal injury, BBB disruption, and neuroinflammation. Several studies have shown that altered levels of brain-enriched proteins in the plasma or serum could be biomarkers of epilepsy and possibly of epileptogenesis [249,250,251]. Some of the most studied biomarkers are as follows: (1) Translocator protein (TSPO), a protein that is expressed on the surface of activated microglia and astrocytes, the main immune cells in the brain. TSPO can be measured by positron emission tomography (PET) imaging, using specific radiotracers that bind to TSPO. TSPO PET imaging can reveal the spatial and temporal profile of neuroinflammation in epilepsy and can also be used to monitor the response to anti-inflammatory treatments [252,253,254,255]. (2) Cytokines and chemokines, small proteins that are secreted by immune cells and other cells and that regulate the inflammatory response. Cytokines and chemokines can be measured in blood or CSF samples, using various methods, such as enzyme-linked immunosorbent assay (ELISA) or multiplex immunoassay. Cytokines and chemokines can reflect the degree and type of neuroinflammation in epilepsy and can also be associated with seizure frequency, severity, and duration [256,257,258]. (3) Bioactive lipids, molecules that are derived from the metabolism of fatty acids, modulate the inflammatory response. Bioactive lipids can be measured in blood or CSF samples, using various methods, such as liquid chromatography–mass spectrometry (LC-MS), gas chromatography–mass spectrometry (GC-MS), or high-performance liquid chromatography (HPLC). Bioactive lipids can indicate the balance between pro-inflammatory and anti-inflammatory pathways in epilepsy and can also be involved in the regulation of neuronal excitability and synaptic plasticity [259,260,261,262].

Biomarkers can help identify at-risk patients and guide the initiation of preventive strategies, such as anti-inflammatory or neuroprotective drugs, before the onset of seizures [263]. However, there is a need for more comprehensive biomarker types, particularly those that can quantitatively understand the overall system and predict differences in disease and treatment outcomes [264]. The identification and validation of biomarkers for epileptogenesis are complex processes, requiring a strategic roadmap and a harmonized pipeline for preclinical multicenter plasma protein and miRNA biomarker discovery [265,266]. These efforts are crucial for the development of effective preventive treatments and the personalization of anti-epileptic drug treatment.

The current state of biomarker research in this field is continually evolving, facing numerous challenges and limitations. The heterogeneity of epilepsy poses a significant obstacle, compounded by variations in the role and extent of neuroinflammation based on factors, such as etiology, location, and stage of epilepsy. Additionally, external influences, including age, sex, comorbidities, and medication, further contribute to the complexity. Therefore, relying on a single biomarker to encapsulate the multifaceted and variable nature of epilepsy is improbable [267]. A more plausible approach involves employing a combination of multiple biomarkers, offering a more informative and accurate representation. These biomarkers must be capable of distinguishing epilepsy from other neurological or systemic disorders involving neuroinflammation, such as Alzheimer’s disease, multiple sclerosis, or rheumatoid arthritis. Moreover, they should be sensitive enough to detect subtle and dynamic changes reflecting various phases of epileptogenesis, including initiation, progression, or maintenance. Advancing this field necessitates the validation of biomarkers in extensive and diverse patient cohorts, measured at multiple time points and under different conditions. Unfortunately, current biomarker research for epileptogenesis faces challenges stemming from fragmented and poorly coordinated efforts. Issues with inter-model reproducibility, study design, and statistical approaches further hinder progress. Validating biomarkers in patients encounters difficulties due to a lack of rigorous characterization and a need for biomarkers in categories beyond diagnosis, such as markers of risk, susceptibility, and prognosis [266,268]. Given the diverse nature of epilepsy, distinct biomarkers might be required for different etiologies. Additionally, the lack of harmonization in sample preparation across centers, poorly characterized phenotypes, and the quality of collected samples poses significant obstacles. Overcoming these challenges demands competence in informatics for handling big data and integrating the use of multimodal biomarkers. Addressing these complexities will be pivotal for advancing reliable biomarkers in the context of epilepsy [269].

Utilizing databases, we can validate biomarkers for epilepsy research by comparing and contrasting data and knowledge from different sources and levels of evidence. Rigorous and robust statistical and computational methods are essential in this process. For instance, biomarkers identified by genetic studies like EpilepsyGene [270] or Epi4K [271] can be validated by confirming their functional relevance and causal role in epilepsy using data from epigenetic, transcriptomic, proteomic, or metabolomic studies, such as EpiGraphDB [272]. Validation efforts can extend to biomarkers associated with specific types or subtypes of epilepsy, testing their specificity and sensitivity in diverse cohorts while adjusting for potential confounders. Predictive or prognostic biomarkers can be validated by assessing their temporal and spatial dynamics and correlation with clinical outcomes, such as seizure frequency, severity, duration, or response to treatment. Therapeutic or preventive biomarkers can undergo validation through evaluation of their pharmacological or biological effects, safety, and efficacy in preclinical and clinical trials. Nevertheless, employing data science techniques introduces its set of challenges and limitations. Addressing these challenges requires data science techniques to uphold ethical and legal principles for data protection, privacy, and consent, ensuring the quality and availability of data through appropriate methods for cleaning, preprocessing, and transformation. Additionally, the techniques must adhere to rigorous standards and guidelines for data modeling, testing, and evaluation [273]. Biomarkers associated with neuroinflammation that could serve as early indicators of epilepsy risk are a valuable and promising research field. Biomarkers can provide insights into the pathophysiology and mechanisms of epilepsy, and they can also facilitate the diagnosis, prognosis, and treatment of epilepsy. However, biomarkers need to be further validated, refined, and integrated to achieve their full potential and clinical utility.

### 5.3. Development of Therapeutic Targets to Modulate Neuroinflammation

The exploration of therapeutic targets to modulate neuroinflammation in scenarios involving viral-induced epilepsy and epilepsy patients acquiring a virus represents a challenging yet promising research frontier. One important approach is the purposeful use of immunomodulator, antiviral, and anti-inflammatory medications.

Antiviral medications play a crucial role in modulating neuroinflammation by mitigating the viral load and components that activate inflammatory pathways [56]. For instance, acyclovir inhibits the DNA polymerase of the herpes simplex virus, reducing viral replication, neuroinflammation, and improving clinical outcomes [274]. Agents inhibiting inflammatory mediators, such as cytokines and chemokines, can reduce neuroinflammation and neuronal damage. Minocycline, an anti-inflammatory drug, suppresses microglial activation and pro-inflammatory cytokine production, thus mitigating neuroinflammation and epileptogenesis [275,276]. These drugs modulate immune cell function, impacting the immune response and immune-mediated damage in the brain. Fingolimod, an immunomodulatory drug, inhibits lymphocyte migration, reducing neuroinflammation and seizure frequency in various viral encephalitis models [277,278]. These effects are attributed to the drug’s ability to retain lymphocytes in lymph nodes, preventing their recruitment to the CNS [279]. However, it is important to note that there have been reports of severe herpes simplex encephalitis reactivation in patients taking fingolimod [280], suggesting a potential risk of viral encephalitis in some cases.

The approach to developing therapeutic targets to modulate neuroinflammation varies based on factors, such as the type and severity of viral infection, the infection’s time and duration, and individual susceptibility and response. Similarities may exist for infections with comparable characteristics, while differences may arise due to distinct routes of neuroinvasion, replication, and clearance. The consideration of individual susceptibility is paramount, with genetic factors influencing inflammatory responses. Tailoring therapeutic targets to individual profiles becomes essential for effective outcomes.

Neuroinflammation in viral-induced epilepsy involves pathways, like TLRs, inflammasomes, and purinergic receptors. Modulating these pathways presents potential therapeutic targets [217]. Modulating these pathways may help control drug-resistant seizures and modify the disease. The IL-1 receptor/TLR signaling, cyclooxygenase-2, tumor necrosis factor-alpha, complement signaling, and chemokines are also implicated in epilepsy and could be potential therapeutic targets [281]. Inhibiting TLR signaling using antagonists or inhibitors demonstrates promise in reducing neuroinflammation and seizure frequency. TLR4 antagonists and NF-κB inhibitors exhibit efficacy in animal models [282,283]. Inhibiting inflammasome activation or IL-1β/IL-18 signaling shows potential, with inflammasome inhibitors and IL-1 receptor antagonists exhibiting positive effects in relevant animal models [284,285]. Furthermore, targeting purinergic P2 receptors, particularly the P2Y1 and P2X7 subtypes, has shown anticonvulsive and anticonvulsant potential [286].

Developing anti-epileptogenic therapies faces challenges due to the heterogeneity of viral infections and epilepsy. Biomarkers and therapeutics must be specific, sensitive, accessible, and feasible. Opportunities lie in technological advancements, collaboration among disciplines, and the integration of innovative methods, like nanotechnology, biotechnology, optogenetics, and artificial intelligence. The need for biomarker identification is emphasized in the pursuit of disease-modifying therapies for epilepsy. Addressing challenges, such as patient variability, ethical concerns, and the necessity for extensive follow-up periods, requires strategic roadmaps and collaborative efforts. The quest for therapeutic targets to modulate neuroinflammation in the context of viral infections and epilepsy presents both challenges and opportunities. A personalized and precision medicine approach, guided by biomarker identification and innovative technologies, holds the potential to significantly impact the diagnosis, prognosis, and treatment of these conditions.

## 6. Clinical Implications

The interactions among viral infections, neuroinflammation, and epilepsy have significant implications for clinical management, presenting manifold challenges [287]. Diagnosing epilepsy and its underlying cause is complex, particularly with viral infections and neuroinflammation, as symptoms can be non-specific, and the temporal relationship is often unclear. Clinicians require a high index of suspicion and use a combination of criteria for diagnosis, relying on clinical, laboratory, neuroimaging, and electrophysiological assessments. Managing epilepsy in the presence of viral infections involves a multidisciplinary approach. Challenges include diagnosing viral infections in epileptic patients, preventing infections due to impaired immunity, and managing infections without compromising epilepsy treatment. Potential drug interactions between antiviral and anticonvulsant drugs pose further challenges, requiring precise therapies tailored to individual conditions [287].

Precision therapies involve selecting appropriate drugs, adjusting doses, monitoring drug levels, and considering adjunctive therapies. The prognosis of epilepsy depends on various factors, including etiology, syndrome, seizure control, and quality of life [288]. Patients may achieve remission or experience persistent or progressive epilepsy. The prognosis of viral infections and neuroinflammation is influenced by factors, like infection severity, brain damage extent, and treatment response, with varying outcomes [289]. Implementing precision therapies faces numerous challenges, such as identifying the precise cause of epilepsy, validating therapy efficacy, and overcoming ethical and regulatory issues. Access to diagnostic tools and therapies can be limited, particularly in low-resource settings [290]. Despite these challenges, there are opportunities to enhance diagnosis, treatment, and prognosis through advances in genomics, immunology, and pharmacology. Collaborative efforts across disciplines, sectors, and regions are crucial for translating scientific knowledge into clinical practice [291]. Patient stratification offers insights for personalized treatment approaches, considering viral infection types, neuroinflammatory profiles, and pharmacokinetic parameters. Stratification enables optimal drug selection and dosing regimens. However, challenges, including the lack of reliable biomarkers and the complexity of processes influencing susceptibility and progression, need addressing in refining patient stratification approaches [292,293].

## 7. Future Directions and Precision Therapies

Based on our growing knowledge of these interactions, the following are some prospective directions for precision therapies: (1) Genetic testing can help to identify the genetic cause of epilepsy, as well as the genetic susceptibility to viral infections or anticonvulsant drugs. This can help to select the most appropriate and effective treatment for each patient, based on their genotype and phenotype. Gene therapy can also offer a promising strategy to correct genetic defects or modify the gene expression that underlies epilepsy or viral infections and to restore the normal function of the brain or the immune system [294,295]. For example, a case report of a neonate with NPRL3-related epilepsy reported 3.5 months of seizure control that allowed the patient to grow seizure-free until epilepsy surgery [296]. (2) Immunotherapy can help to modulate the immune response to viral infections or anticonvulsant drugs and to prevent or treat the neuroinflammation that can trigger or worsen seizures and epilepsy. Immunotherapy can include the use of vaccines, antibodies, cytokines, or immunomodulators, depending on the type and stage of the viral infection or the epilepsy. Anti-inflammatory drugs can also help to reduce the inflammation and edema that can damage the brain or disrupt the blood–brain barrier and restore the balance between the excitatory and inhibitory neurotransmitters and receptors [285,297,298]. For example, a review of targeted therapies for monogenic epilepsy syndromes suggested that anti-inflammatory drugs, such as steroids or non-steroidal anti-inflammatory drugs, may be beneficial for patients with epilepsy due to mutations in genes involved in the innate immune system, such as NLRP3 or NLRC41 [299]. (3) Neuromodulation and neurostimulation can help to regulate the electrical activity and connectivity of the brain networks that are involved in seizures and epilepsy and to prevent or abort the seizure onset or propagation. Neuromodulation and neurostimulation can include the use of devices, such as vagus nerve stimulators, deep brain stimulators, or responsive neurostimulators, that can deliver electrical impulses to specific brain regions or nerves, either continuously or in response to seizure detection. Neuromodulation and neurostimulation can also include the use of non-invasive techniques, such as transcranial magnetic stimulation or transcranial direct-current stimulation, that can modulate cortical excitability or plasticity [300,301]. For example, a study using precision medicine for the diagnosis and treatment of viral infections and epilepsy suggested that neuromodulation and neurostimulation may be useful for patients with refractory epilepsy who are not candidates for surgery or who have contraindications to antiviral drugs or anticonvulsant drugs [302].

Some areas that need more research, as well as some current and developing technology, are as follows: Biomarkers and biosensors can help to improve the diagnosis, prognosis, and monitoring of viral infections and epilepsy and to guide the treatment decisions and adjustments. Biomarkers and biosensors can include the use of molecular, genetic, immunological, or metabolic markers that can indicate the presence, severity, or progression of viral infections or epilepsy or the response or resistance to antiviral drugs or anticonvulsant drugs. Biosensors can also include the use of wearable or implantable devices that can measure the physiological, behavioral, or environmental parameters that can predict, detect, or influence the occurrence or outcome of viral infections or epilepsy. For example, a review of new trends and the most promising therapeutic strategies for epilepsy treatment suggested that biomarkers and biosensors may be useful for identifying the optimal timing and duration of treatment and for preventing or reducing the adverse effects or complications of viral infections or epilepsy.

Artificial intelligence and machine learning can help to analyze the large and complex data sets that are generated by the various sources of information, such as genomic, transcriptomic, proteomic, metabolomic, neuroimaging, electrophysiological, or clinical data, and to extract meaningful and relevant patterns or insights that can enhance the understanding and management of viral infections and epilepsy. Artificial intelligence and machine learning can also help to develop and validate the predictive models or algorithms that can forecast the risk, occurrence, or outcome of viral infections or epilepsy or the response or resistance to antiviral drugs or anticonvulsant drugs and to provide personalized and optimized recommendations or interventions for each patient. For example, a review of seizures and epilepsy secondary to viral infection in the central nervous system suggested that artificial intelligence and machine learning may be useful for identifying the optimal anticonvulsant drugs or antiviral drugs for each patient and for improving the seizure detection or prediction.

Stem cell therapy and tissue engineering can help to repair or replace the damaged or lost brain cells or tissues that are caused by viral infections or epilepsy and to restore the normal structure and function of the brain or the immune system. Stem cell therapy and tissue engineering can include the use of various types of stem cells, such as embryonic, induced pluripotent, or neural stem cells that can differentiate into neurons, glia, or other cell types, and that can be delivered to the brain by injection, transplantation, or implantation. Tissue engineering can also include the use of scaffolds, matrices, or bioreactors that can support the growth, survival, or integration of the stem cells or the engineered tissues. For example, a review of viral infections and epilepsy exploring causality suggested that stem cell therapy and tissue engineering may be useful for patients with epilepsy due to viral infections that cause extensive brain damage or scarring, such as herpes simplex virus or rabies virus.

## 8. Conclusions

Epilepsy and viral infections are two common and complex conditions that can affect the central nervous system and cause significant morbidity and mortality. The relationship between epilepsy and viral infections is bidirectional and multifactorial, as viral infections can induce or exacerbate seizures and epilepsy, and epilepsy patients can acquire or worsen viral infections. The mechanisms underlying the interaction between epilepsy and viral infections involve various factors, such as the type and severity of the infection and epilepsy, the genetic and immunological susceptibility of the host, the pharmacological and pharmacokinetic properties of the antiviral and anticonvulsant drugs, and the role of neuroinflammation and neuroimmunity in modulating the neuronal function and viability. The diagnosis and treatment of epilepsy and viral infections require a comprehensive and multidisciplinary approach, which includes the identification and management of the etiological agents, the prevention and control of the seizures and the infection, the monitoring and adjustment of the drug levels and effects, and the evaluation of and improvement in the clinical outcomes and the quality of life. The research and development of new and effective therapies and vaccines for epilepsy and viral infections are also essential to reduce the burden and the impact of these conditions on the individual and society.

## Figures and Tables

**Table 1:** Distinctions and commonalities between epilepsy patients acquiring viral infection and individuals developing epilepsy after viral infection.

Considerations	Epilepsy Patients Acquiring Viral Infection	Individuals Developing Epilepsy after Viral Infection
Type and severity of the viral infection	Epilepsy patients acquiring viral infections may exhibit diverse types and severities, influenced by their brain and immune system susceptibility. Examples include severe or chronic infections like human immunodeficiency virus (HIV), cytomegalovirus, or Epstein-Barr virus, leading to persistent brain infection, chronic neuroinflammation, and seizures.	Individuals developing epilepsy after viral infection may experience less severe or acute infections, such as herpes simplex virus, influenza virus, or severe acute respiratory syndrome -coronavirus-2 (SARS-CoV-2). These infections may cause transient brain infection, acute neuroinflammation, and seizures.
Time and duration of the infection	Epilepsy patients acquiring viral infections may have varying infection timelines and durations, depending on the pre-existing epilepsy status. Viral infections may exacerbate or modify existing epilepsy.	Individuals developing epilepsy after viral infection may lack a history of epilepsy before the viral event. The infection may serve as an initiator or trigger, influencing the onset and progression of epilepsy.
Individual susceptibility and response to the infection	Epilepsy patients acquiring viral infections may display distinct susceptibilities and responses, influenced by genetic, environmental, and immunological factors. Genetic factors, like polymorphisms or mutations in inflammatory genes, may modulate the response and epilepsy susceptibility.	Individuals developing epilepsy after viral infection may be influenced by environmental factors (age, sex, comorbidities, or medication) that modulate the inflammatory response and susceptibility to epilepsy. Immunological factors, including the immune response type and magnitude, balance between pathways, and resolution, may also play a role.

## Data Availability

No new data were created or analyzed in this study. Data sharing is not applicable to this article.
